# The study on serum oxidized low-density lipoprotein and homocysteine as cardiovascular risk markers in subclinical hypothyroidism patients

**DOI:** 10.3389/fendo.2026.1750486

**Published:** 2026-01-23

**Authors:** Qian Wang, Xiaonyun Zhang, Fan Wu, Xinkun Qi

**Affiliations:** 1 Department of Medical Laboratory, Fuwai Central China Cardiovascular Hospital/Central China Fuwai Hospital of Zhengzhou University, Zhengzhou, Henan, China; 2 Neurology Laboratory of the First Affiliated Hospital of Zhengzhou University, Zhengzhou, Henan, China

**Keywords:** cardiovascular risk factors, homocysteine, oxidized low-density lipoprotein, subclinical hypothyroidism, thyroid-stimulating hormone

## Abstract

**Purpose:**

Hypothyroidism, the most prevalent endocrine disorder globally, is associated with increased cardiovascular risk. This study aims to evaluate cardiovascular risk factors—including serum oxidized low-density lipoprotein (ox-LDL), serum homocysteine (Hcy), and lipid profiles—and their correlations with thyroid-stimulating hormone (TSH) levels. Early identification of these risk predictors may reduce the incidence and mortality of cardiovascular disease in hypothyroid patients.

**Patients and methods:**

This cross-sectional study included 676 participants. Subjects were stratified into four groups: three corresponding to TSH quartiles within the reference range and a fourth comprising subclinical hypothyroidism (SCH) patients with TSH levels above this range. All participants underwent physical examinations and provided fasting blood samples for measurement of TSH, free thyroxine (FT4), free triiodothyronine (FT3), blood glucose, triglycerides (TG), total cholesterol (TC), high-density lipoprotein cholesterol (HDL-C), low-density lipoprotein cholesterol (LDL-C), apolipoprotein A1 (ApoA1), apolipoprotein B (ApoB), lipoprotein(a) [Lp(a)], ox-LDL, and Hcy.

**Results:**

Across the four subgroups, LDL-C, ApoB, ox-LDL, and Hcy levels exhibited significant increasing trends (all *p <* 0.05; specific *p =* 0.01, *p =* 0.01, *p <* 0.01, *p <* 0.01, respectively), whereas HDL-C decreased significantly (*p <* 0.01). Specifically, compared to the T1 subgroup (TSH: 0.27-1.58 mIU/L), the SCH subgroup (TSH ≥ 4.20 mIU/L) had significantly higher levels of ox-LDL (1.78 ± 0.49 ng/mL vs. 1.05 ± 0.68 ng/mL, *p <* 0.01) and Hcy (9.87 (interquartile range (IQR): 8.45-11.42) μmol/L vs. 9.22 (IQR: 8.11-10.11) μmol/L, *p <* 0.01), and lower levels of HDL-C (1.29 ± 0.36 mmol/L vs. 1.43 ± 0.39 mmol/L, *p <* 0.01). After adjusting for age and sex, TSH levels demonstrated positive correlations with body mass index (BMI), triglycerides, total cholesterol, LDL-C, ApoB, ox-LDL, and Hcy (all *p <* 0.05), and a negative correlation with HDL-C (*p =* 0.01). Multiple linear regression analysis revealed that TSH levels were independently associated with elevated ox-LDL (β = 0.18, *p <* 0.01) and Hcy (β = 0.11, *p <* 0.01), and reduced HDL-C (β = −0.16, *p =* 0.01).

**Conclusion:**

The observed correlations between ox-LDL, Hcy, and dyslipidemia in subclinical hypothyroidism may indicate a proatherogenic state. Elevated ox-LDL and Hcy emerge as independent factors associated with accelerated atherosclerosis in this condition.

## Introduction

Hypothyroidism is a prevalent endocrine disorder characterized by deficient thyroid hormone production, exhibiting a spectrum from overt disease to the more frequently encountered subclinical form (SCH). Epidemiological studies consistently demonstrate a significant female predominance (female-to-male ratio up to 4-5:1) and a clear increase in incidence with advancing age, particularly after 60 years (1–4). In China, the overall prevalence of hypothyroidism in adults is approximately 9.3%, with the prevalence of SCH being notably higher at 8.7% compared to 1.1% for the overt form. Furthermore, regional variations exist, with higher prevalence rates reported in northwestern China (5).

A substantial body of evidence has firmly established subclinical hypothyroidism as an independent risk factor for the development of cardiovascular disease (CVD) and associated mortality. The risk extends beyond SCH, as even within the conventional euthyroid reference range, a higher thyroid-stimulating hormone (TSH) level is associated with an adverse cardiovascular risk profile (6–8). This suggests a continuum of risk correlating with TSH concentrations, although the precise pathophysiological mechanisms underlying this association remain incompletely elucidated (9, 10).

It is hypothesized that these mechanisms may involve direct effects on vascular endothelial function and systemic metabolic alterations. Dyslipidemia is a common feature, with SCH patients often exhibiting elevated levels of total cholesterol (TC), low-density lipoprotein cholesterol (LDL-C), and triglycerides (TG) (11). Furthermore, elevated homocysteine (Hcy), a marker of endothelial dysfunction and thrombotic risk, is frequently observed in these individuals (12). Notably, both oxidized low-density lipoprotein (ox-LDL) and Hcy contribute to endothelial dysfunction and promote inflammatory processes that accelerate atherosclerosis. ox-LDL facilitates foam cell formation and plaque instability, while Hcy induces oxidative stress and impairs nitric oxide bioavailability (13, 14). Consequently, there is growing interest in the role of other emerging biomarkers integral to atherogenesis, such as ox-LDL, a key driver of plaque formation; apolipoproteins (ApoA1 and ApoB), which provide a nuanced assessment of cardiovascular risk; and lipoprotein(a) [Lp(a)], a potent independent genetic risk factor. Despite their established roles in atherosclerosis, ox-LDL and Hcy are not routinely screened in clinical practice. This underscores the need for studies like ours to evaluate their utility in risk stratification.

It is important to note that SCH seldom occurs in isolation. It is frequently intertwined with components of the cardio-metabolic-renal (CMR) syndrome, including insulin resistance, hypertension, and dyslipidemia, suggesting shared underlying pathways of chronic inflammation and endothelial dysfunction (15). The high prevalence of SCH and its metabolic sequelae, along with the potential cardiovascular impact of high-normal TSH levels, make a thorough investigation crucial. Therefore, this study aims to systematically investigate the associations between serum TSH levels—across the spectrum from high-normal euthyroidism to overt hypothyroidism—and these promising cardiovascular risk markers, including ox-LDL, Hcy, and a comprehensive lipid/apolipoprotein profile. Our study also contextualizes these findings within the broader framework of oxidative stress and compares them with similar investigations in diverse populations. We posit that elucidating these correlations will provide critical insights into the potential mechanistic links between thyroid function and cardiovascular homeostasis, identifying novel associations that may inform future investigative and clinical strategies. Ultimately, timely assessment of these novel indicators in patients with (or at risk of) thyroid dysfunction could facilitate earlier, more precise risk stratification. This, in turn, would enable targeted interventions aimed at reducing the substantial cardiovascular morbidity and mortality in this population.

## Materials and methods

### Study population

**Figure 1:**
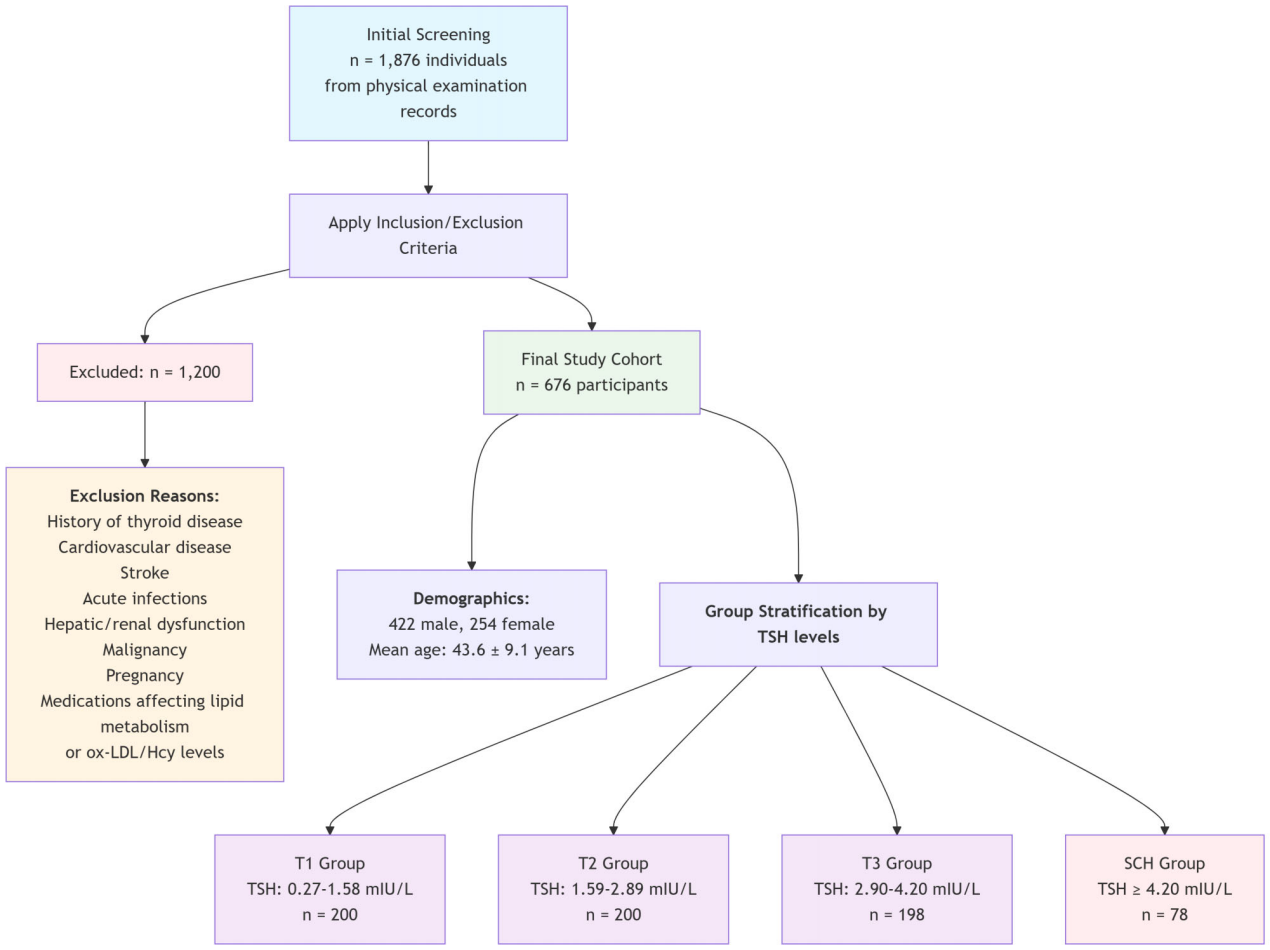
Flowchart of patient selection. SCH, subclinical hypothyroidism; ox-LDL, oxidized low-density lipoprotein; Hcy, serum homocysteine.

This retrospective cross-sectional study initially screened 1876 individuals from physical examination records at Fuwai Central China Cardiovascular Hospital. Inclusion required euthyroid status (free triiodothyronine (FT3): 3.10-6.80 pmol/L; free thyroxine (FT4): 12.00-22.00 pmol/L) with TSH levels either within (0.27-4.20 mIU/L) or above the reference range. Exclusion criteria comprised: history of thyroid disease, cardiovascular disease, stroke, acute infections, hepatic/renal dysfunction, malignancy, pregnancy, or use of medications affecting lipid metabolism or ox-LDL/Hcy levels. Due to the retrospective design, data on several lifestyle and dietary factors—including smoking status, alcohol consumption, physical activity levels, inflammatory markers (e.g., C-reactive protein, interleukin-6), and specific nutrient levels (e.g., vitamin B6, B12, folate)—were not systematically collected and thus not included in the adjustment models. The final cohort comprised 676 participants (422 male, 254 female; mean age 43.6 ± 9.1 years). The study flowchart is depicted in **Figure 1**. The sample sizes across TSH-based subgroups reflect the natural distribution of thyroid function in the screened population, with a smaller proportion meeting criteria for subclinical hypothyroidism, consistent with its epidemiological prevalence. While this results in unequal group sizes, all statistical methods applied were chosen to appropriately handle such imbalance. This study strictly adhered to the Helsinki Declaration and was approved by the local institutional ethics committee.

### Data collection

All participants underwent a standardized physical examination. Height and weight were measured in light indoor clothing without shoes to calculate body mass index (BMI). BMI was calculated as weight in kilograms divided by height in meters squared (kg/m²). It served as the primary anthropometric measure in this study due to its consistent availability in the retrospective health records. We acknowledge that more specific measures of adiposity and body composition, such as waist circumference or body fat percentage—which may be particularly informative for cardiometabolic risk assessment in Asian populations—were not systematically available in this dataset. Seated blood pressure was measured twice on the right arm after 5 minutes of rest using an automated device, with the average recorded. History of hypertension and diabetes was obtained by questionnaire and cross-verified with medical records.

Fasting venous blood samples were collected from all participants in the early morning (between 7:00 and 9:00 AM) after an 8–12 hour overnight fast. Serum was separated by centrifugation at 3000 × g for 10 minutes at room temperature, immediately aliquoted into cryogenic tubes, and stored at -80°C. To ensure analyte stability (particularly for ox-LDL) and minimize batch variation, all samples were analyzed in a single dedicated assay run after sample collection was complete. This aliquoting strategy prevented repeated freeze-thaw cycles, with each aliquot being thawed only once for the analysis. Serum ox-LDL concentrations were quantified using a commercially available sandwich enzyme-linked immunosorbent assay (ELISA) kit (Shanghai Shenggong Technology, Catalog#: E-EL-0120), strictly according to the manufacturer’s protocol. The assay’s performance characteristics, as reported by the manufacturer and verified in our laboratory during assay setup using control samples, include a detection range of 0.31–20 ng/mL, a sensitivity of 0.19 ng/mL, an intra-assay coefficient of variation (CV) < 8%, and an inter-assay CV < 10%. In our study, internal quality control samples (low, medium, and high concentrations) were included in each plate to monitor assay performance. All samples, standards, and controls were assayed in duplicate, and the mean value was used for analysis. The average intra-assay CV for duplicate measurements in our study cohort was 5.2%. Serum concentrations of Hcy, FT3, FT4, TSH, TG, TC, HDL-C, LDL-C, ApoA1, ApoB, and Lp(a) were measured on a Roche Cobas 8000 analyzer according to manufacturer protocols. All assays were performed according to the manufacturer’s protocols, with routine quality control procedures followed. The analytical performance for key assays was as follows: Hcy (CV < 4%), TSH (CV < 5%), LDL-C (CV < 3%).

### Classification based on thyroid condition

Participants were stratified into four analytical groups: three euthyroid subgroups partitioned by TSH reference range quartiles (0.27-4.20 mIU/L) - T1 (0.27-1.58 mIU/L), T2 (1.59-2.89 mIU/L), and T3 (2.90-4.20 mIU/L) - and a fourth SCH group defined as TSH ≥ 4.20 mIU/L with normal FT3/FT4 levels.

### Statistical analysis

Data were managed in Excel and analyzed using SPSS Statistics 27.0 (IBM Corp.). Normally distributed continuous variables are presented as mean ± standard deviation and compared using one-way ANOVA. Non-normally distributed variables are expressed as median (Q1, Q3) and analyzed following logarithmic or arctangent transformation. Categorical data are reported as frequencies (percentages) with between-group comparisons assessed by χ² tests. Given the unequal sample sizes across groups, non-parametric tests and regression models robust to heteroscedasticity were employed where appropriate to ensure valid comparisons. Spearman’s rank correlation evaluated associations between thyroid hormones and cardiovascular risk factors. Univariate logistic regression analyzed relationships between TSH levels and cardiovascular risk factors, while multivariate linear regression identified variables independently associated with TSH levels. The multivariate models were adjusted for age, sex, BMI, hypertension, diabetes status, and estimated glomerular filtration rate (eGFR) to control for potential confounding effects. Statistical significance was defined as *p <* 0.05. To assess potential effect modification by sex, supplementary analyses were performed: (1) key comparative and correlation analyses were stratified by sex; and (2) a multiplicative interaction term (TSH × Sex) was included in the primary multiple linear regression model to formally test for interaction effects on the main outcomes.

## Results

### General characteristics and hormone level comparison of TSH subgroups

This study enrolled 676 participants. The overall prevalence of SCH was 11.5% (78 of 676). Prevalence rates were 10.2% (43 of 422) in males and 13.8% (35 of 254) in females. The general clinical characteristics and hormone levels of the participants are presented in **Table 1**. As expected, FT3 and FT4 levels were significantly decreased across the four groups (classified by thyroid function status), with the lowest levels observed in the SCH group (*p =* 0.01). The proportion of female patients exhibited an increasing trend across the groups and was highest in the SCH group (*p <* 0.01). No statistically significant difference in age was observed between the groups.

**Table 1:** General clinical characteristics and hormone level comparison of TSH subgroups.

Variables	T1 (n =200)	T2 (n = 200)	T3 (n = 198)	SCH (n = 78)	p
Gender (Male/Female)	132/68	121/79	126/72	43/35	<0.01
Age (years)	42.78 ± 9.98	43.74 ± 8.59	47.23 ± 8.37	45.65 ± 9.76	0.56
TSH/(mIU/L)	1.09(0.87,1.42)	1.97(1.79,2.78)	3.46(3.02,3.86)	5.43(4.65,6.78)	<0.01
FT3/(pmol/L)	4.79 ± 0.78	4.69 ± 0.82	4.63 ± 0.67	4.52 ± 0.75	0.01
FT4/(pmol/L)	17.78 ± 2.13	17.52 ± 2.34	17.12 ± 2.26	15.94 ± 2.08	0.01

SCH, subclinical hypothyroidism; TSH, thyroid-stimulating hormone; FT3, free triiodothyronine; FT4, free thyroxine.

### Comparison of cardiovascular risk factors between TSH subgroups

**Figure 2:**
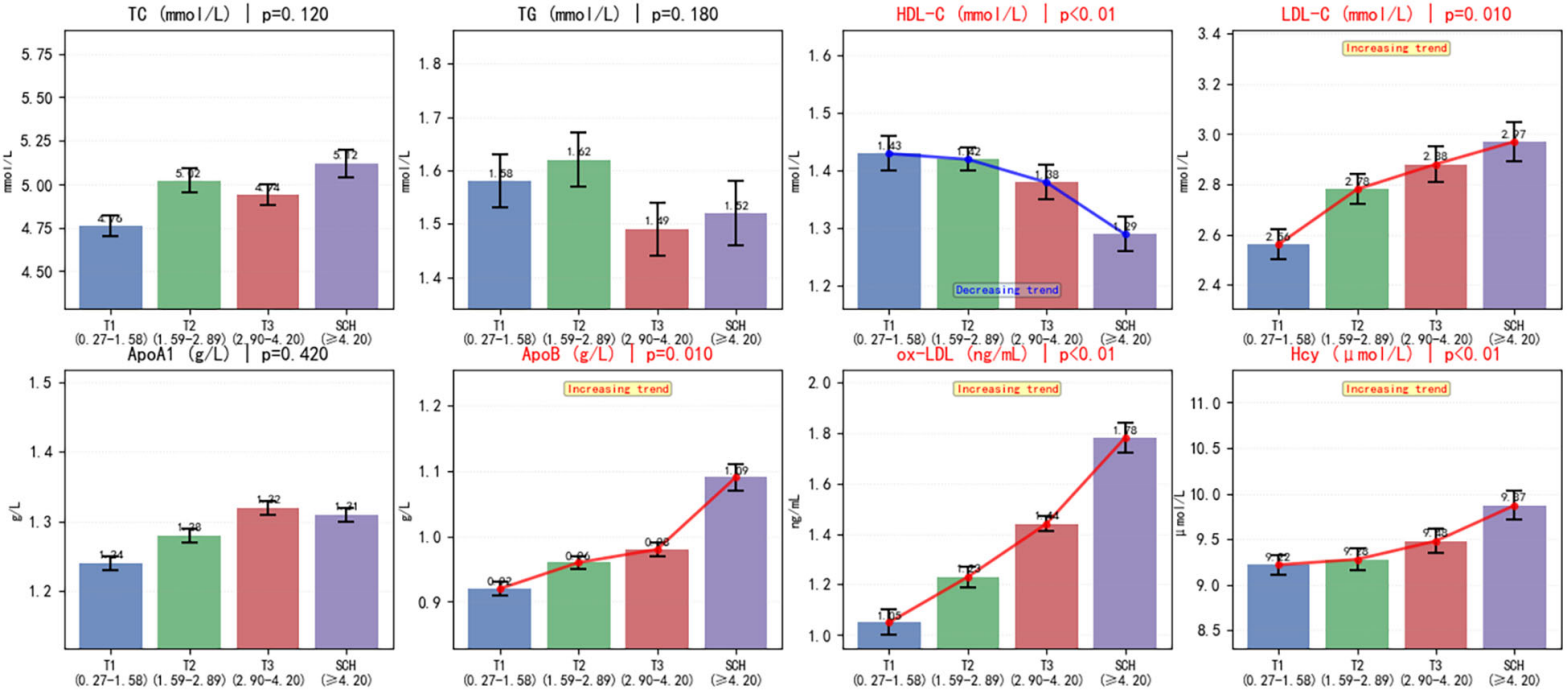
Comprehensive biomarker profiles across TSH subgroups in subclinical hypothyroidism. TC, total cholesterol; TG, triglycerides; HDL-C, high-density lipoprotein cholesterol; LDL-C, low-density lipoprotein cholesterol; ApoA1, apolipoprotein A1; ApoB, apolipoprotein B.

The comprehensive analysis of cardiovascular risk factors across the four TSH-defined subgroups revealed significant variations in multiple biomarkers, as detailed in **Table 2** and visually represented in **Figure 2**. Among the conventional lipid parameters, HDL-C demonstrated a significant decreasing trend from T1 (1.43 ± 0.39 mmol/L) to SCH (1.29 ± 0.36 mmol/L), which remained highly significant after FDR correction (unadjusted *p <* 0.001, adjusted *q* = 0.004). LDL-C showed an increasing pattern from T1 (2.56 ± 0.91 mmol/L) to SCH (2.97 ± 0.86 mmol/L), with nominal significance (unadjusted *p =* 0.01) that approached but did not reach the threshold after FDR correction (adjusted *q* = 0.048). Similarly, ApoB exhibited a progressive increase (T1: 0.92 ± 0.14 g/L to SCH: 1.09 ± 0.16 g/L) with unadjusted *p =* 0.01 and adjusted *q* = 0.048. The most pronounced alterations were observed in the emerging cardiovascular risk markers. ox-LDL demonstrated a striking dose-dependent elevation across TSH strata, with levels increasing significantly from T1 (1.05 ± 0.68 ng/mL) to SCH (1.78 ± 0.49 ng/mL), remaining highly significant after FDR correction (unadjusted *p <* 0.001, adjusted *q* = 0.004). Similarly, Hcy concentrations showed a significant progressive rise from T1 (9.22 [8.11-10.11] μmol/L) to SCH (9.87 [8.45-11.42] μmol/L; unadjusted *p <* 0.001, adjusted *q* = 0.004). The comprehensive biomarker profiles illustrated in **Figure 2** visually reinforce these statistical trends, particularly highlighting the significant increases in LDL-C, ApoB, ox-LDL, and Hcy, alongside the decreasing HDL-C pattern across the four TSH subgroups. No significant differences were observed among groups for hypertension prevalence, diabetes prevalence, body mass index, or Lp(a) levels.

**Table 2:** Comparison of traditional and new cardiovascular risk factors between TSH subgroups.

Variables	T1 (n =200)	T2 (n = 200)	T3 (n = 198)	SCH (n = 78)	p-value	FDR-adjusted q
Hypertension(%)	19.45	15.78	17.43	16.29	0.54	0.730
Diabetes(%)	28.56	30.26	30.76	32.89	0.48	0.720
BMI/(kg/m2)	22.89 ± 1.89	22.12 ± 1.88	23.02 ± 1.67	22.79 ± 1.82	0.44	0.704
TC/(mmol/L)	4.76 ± 0.91	5.02 ± 1.05	4.94 ± 0.89	5.12 ± 1.12	0.12	0.360
TG/(mmol/L)	1.58(0.98,2.03)	1.62(1.04,1.99)	1.49(1.01,2.09)	1.52(0.94,2.12)	0.18	0.432
HDL-C/(mmol/L)	1.43 ± 0.39	1.42 ± 0.32	1.38 ± 0.45	1.29 ± 0.36	<0.01	0.004
LDL-C/(mmol/L)	2.56 ± 0.91	2.78 ± 0.89	2.88 ± 0.99	2.97 ± 0.86	0.01	0.048
ApoA1/(g/L)	1.24 ± 0.14	1.28 ± 0.16	1.32 ± 0.19	1.31 ± 0.15	0.42	0.672
ApoB/(g/L)	0.92 ± 0.14	0.96 ± 0.18	0.98 ± 0.11	1.09 ± 0.16	0.01	0.048
Lp(a)(mg/dL)	12.5(5.6-22.8)	13.2(6.8-20.2)	14.2(7.8-22.1)	12.2(7.9-24.5)	0.73	0.876
Ox-LDL(ng/mL)	1.05 ± 0.68	1.23 ± 0.56	1.44 ± 0.38	1.78 ± 0.49	<0.01	0.004
Hcy(µmol/L)	9.22(8.11,10.11)	9.28(8.34,10.91)	9.48(8.25,11.22)	9.87(8.45,11.42)	<0.01	0.004

BMI, body mass index; TC, total cholesterol; TG, triglycerides; HDL-C, high-density lipoprotein cholesterol; LDL-C, low-density lipoprotein cholesterol; ApoA1, apolipoprotein A1; ApoB, apolipoprotein B; Lp(a), lipoprotein(a); ox-LDL, oxidized low-density lipoprotein; Hcy, serum homocysteine.

Data are expressed as mean ± standard deviation or median (interquartile range). The p-values were obtained from one-way ANOVA or the Kruskal-Wallis test, as appropriate. To control the false discovery rate arising from multiple comparisons, q-values were adjusted using the Benjamini-Hochberg procedure, with a q-value < 0.05 deemed statistically significant.

### Univariate logistic regression analysis

To further assess the unadjusted associations between cardiovascular risk factors and the likelihood of having a higher TSH level, univariate logistic regression was performed (**Table 3**). TSH was dichotomized at the median value of the study population for this analysis. Consistent with the trends observed in **Table 2**, several factors were significantly associated with higher odds of being in the elevated TSH group. These included lower HDL-C (odds ratio (OR) = 0.65, 95% CI: 0.48-0.88, *p <* 0.01), and higher levels of LDL-C (OR = 1.28, 95% CI: 1.06-1.55, *p =* 0.01), ApoB (OR = 1.35, 95% CI: 1.10-1.66, *p <* 0.01), ox-LDL (OR = 1.82, 95% CI: 1.45-2.28, *p <* 0.01), and Hcy (OR = 1.15, 95% CI: 1.05-1.26, *p <* 0.01).

**Table 3:** Univariate logistic regression analysis of cardiovascular risk factors with TSH level.

Variable	Odds ratio (OR)	95% Confidence interval	p-value
Hypertension	1.12	0.87 - 1.44	0.38
Diabetes	1.08	0.82 - 1.42	0.59
BMI (per 1 kg/m²)	1.04	0.98 - 1.10	0.19
TC (per 1 mmol/L)	1.15	0.96 - 1.38	0.13
TG (per 1 mmol/L)	1.05	0.90 - 1.22	0.55
HDL-C (per 1 mmol/L)	0.65	0.48 - 0.88	<0.01
LDL-C (per 1 mmol/L)	1.28	1.06 - 1.55	0.01
ApoA1 (per 1 g/L)	0.92	0.78 - 1.09	0.34
ApoB (per 1 g/L)	1.35	1.10 - 1.66	<0.01
Lp(a) (per 10 mg/dL)	1.02	0.99 - 1.05	0.18
ox-LDL (per 1 ng/mL)	1.82	1.45 - 2.28	<0.01
Hcy (per 1 μmol/L)	1.15	1.05 - 1.26	<0.01

### Correlation analysis between thyroid hormones and cardiovascular risk factors

Partial correlation analysis, adjusted for age and sex, was performed to examine the relationships between thyroid hormones and cardiovascular risk markers in the entire cohort. After adjustment, TSH levels demonstrated significant positive correlations with BMI, TC, TG, LDL-C, ApoB, ox-LDL, and Hcy (all *p <* 0.05), and a significant negative correlation with HDL-C (*p <* 0.01). Conversely, FT4 levels showed significant negative correlations with TC, LDL-C, ApoB, ox-LDL, and Hcy (all *p <* 0.05). FT3 levels were positively correlated with TG and HDL-C (*p <* 0.05) (**Table 4**).

**Table 4:** The relationship between thyroid hormones and cardiovascular risk factors.

Variable	FT3		FT4		TSH	
	r	p	r	p	r	p
Hypertension	0.07	0.14	0.02	0.53	0.08	0.06
Diabetes	0.03	0.82	0.11	0.34	0.29	0.09
BMI	0.07	0.25	-0.07	0.44	0.09	0.04
TC	0.08	0.06	-0.08	0.02	0.06	0.03
TG	0.08	0.04	-0.02	0.44	0.04	0.01
HDL-C	0.11	0.01	0.05	0.15	-0.18	0.01
LDL-C	0.12	0.06	-0.07	0.04	0.11	<0.01
ApoA1	-0.08	0.29	-0.12	0.09	-0.15	0.07
ApoB	0.15	0.09	-0.02	0.02	0.10	<0.01
Lp(a)	0.06	0.47	0.14	0.08	0.08	0.09
ox-LDL	0.08	0.07	-0.06	0.01	0.15	<0.01
Hcy	0.06	0.08	-0.12	<0.01	0.12	<0.01

**Figure 3:**
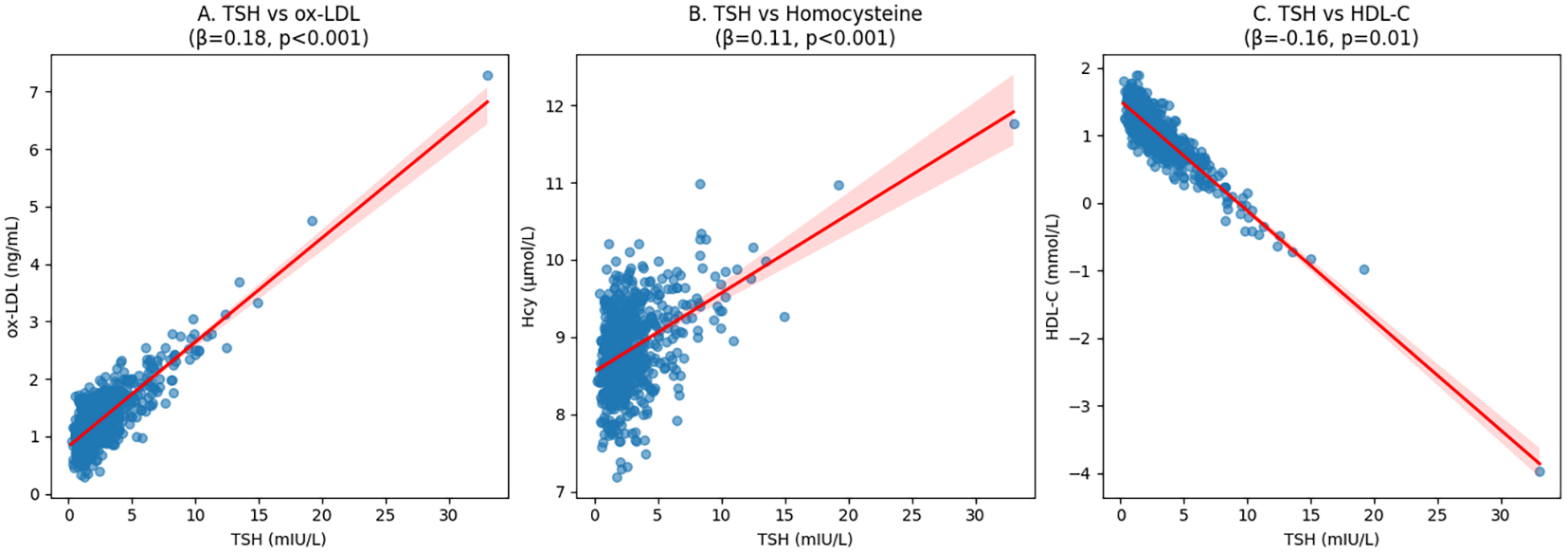
Correlation between TSH levels and cardiovascular risk biomarkers.

Scatter plot analysis revealed significant linear relationships between TSH levels and key cardiovascular risk biomarkers, as illustrated in **Figure 3**. TSH demonstrated strong positive correlations with both oxidized LDL (ox-LDL; β = 0.18, *p <* 0.01) and homocysteine (Hcy; β = 0.11, *p <* 0.01), indicating that higher TSH levels were associated with increased oxidative stress and thrombotic risk markers. Conversely, a significant negative correlation was observed between TSH and HDL-C (β = -0.16, *p =* 0.01), suggesting an inverse relationship with cardioprotective lipids. The strength of these associations was particularly notable for ox-LDL, which showed the steepest regression slope among all biomarkers analyzed. These correlation patterns persisted after adjustment for age and sex, supporting the robustness of the observed relationships.

### Analysis of independent variables related to TSH

Multiple linear regression analysis was performed to identify factors independently associated with TSH levels. The model included sex, age, BMI, prevalence of hypertension, prevalence of diabetes, TG, TC, HDL-C, LDL-C, ApoA1, ApoB, Lp(a), ox-LDL, Hcy, FT3, and FT4 as independent variables. After adjustment for covariates, TSH levels were independently and positively associated with female sex (β = 0.32, *p <* 0.01), ox-LDL (β = 0.18, *p <* 0.01), and Hcy (β = 0.11, *p <* 0.01), while showing a negative association with HDL-C (β = -0.16, *p =* 0.01) (**Table 5**).

**Table 5:** Multiple linear regression analysis of independent variables related to TSH levels.

Variables	β	t	p
Female	0.32	3.75	<0.01
ox-LDL	0.18	2.78	<0.01
Hcy	0.11	2.42	<0.01
HDL-C	-0.16	-3.92	0.01

The model was adjusted for age, sex, BMI, hypertension, diabetes, TG, TC, LDL-C, ApoA1, ApoB, Lp(a), FT3, and FT4.

### Exploratory analyses by sex and interaction testing

Given the marked female predominance in thyroid disorders and potential sex differences in cardiovascular risk profiles, we conducted supplementary analyses to explore whether the observed associations varied by sex. First, we stratified the cohort by sex (422 males, 254 females) and repeated the comparative analysis of cardiovascular biomarkers across TSH subgroups. The direction of adverse trends—increasing ox-LDL, Hcy, and LDL-C, and decreasing HDL-C with rising TSH—was consistent in both males and females. For instance, in the female subgroup, median Hcy levels increased from 8.95 [IQR: 7.88-9.81] μmol/L in the T1 group to 10.12 [8.61-11.98] μmol/L in the SCH group (*p <* 0.01). In males, the corresponding increase was from 9.41 [8.23-10.45] μmol/L to 9.76 [8.32-11.15] μmol/L (*p =* 0.02).

To formally test for effect modification by sex, we extended the primary multiple linear regression model (the model presented in **Table 5**) by including a multiplicative interaction term (TSH × Sex). The results indicated no statistically significant interaction effect of sex on the associations between TSH and the key biomarkers: for ox-LDL (β for interaction = 0.04, 95% CI: -0.10 to 0.18, *p =* 0.58), for Hcy (β = 0.03, 95% CI: -0.08 to 0.14, *p =* 0.61), and for HDL-C (β = -0.05, 95% CI: -0.15 to 0.05, *p =* 0.32). This indicates that the strength and direction of the relationships between TSH levels and these cardiovascular risk markers did not differ significantly between men and women in our study population. The detailed results of the sex-stratified analyses are presented in **Supplementary Table 1**.

## Discussion

This retrospective study demonstrated that levels of LDL-C, ApoB, ox-LDL, and Hcy increased with rising TSH levels, whereas HDL-C levels decreased. Furthermore, significant associations were observed between TSH levels and multiple cardiovascular risk factors. Multiple linear regression analysis confirmed that TSH levels were independently and positively associated with ox-LDL and Hcy, and negatively associated with HDL-C. These findings suggest that individuals with SCH or high-normal TSH levels may be at increased cardiovascular risk. However, the cross-sectional nature of this study precludes causal inference, and the observed associations should be interpreted with caution.

In this study, ox-LDL and Hcy levels were significantly elevated in SCH patients (*p <* 0.01). These biomarkers are integral to the pathogenesis of atherosclerosis through intertwined pathways. Elevated serum Hcy levels have been consistently observed in patients with newly diagnosed hypothyroidism compared to healthy controls (*p <* 0.01) (9, 16). Furthermore, Boushey et al. demonstrated that a 4 μmol/L increase in Hcy levels relative to healthy individuals is associated with a 40% increase in coronary heart disease risk, strongly suggesting that elevated serum Hcy may contribute to higher cardiovascular risk (17). Mechanistically, hyperhomocysteinemia (HHcy) promotes endothelial dysfunction by reducing the availability of nitric oxide, stimulating vascular smooth muscle cell proliferation, and enhancing oxidative stress (13). During inflammation, inflammatory cells release Hcy, contributing to HHcy. HHcy, often linked to cystathionine β-synthase dysfunction, is an established independent risk factor for cardiovascular disease, particularly coronary artery disease (18, 19). HHcy is associated with processes that may accelerate atherosclerosis, such as the promotion of LDL-C oxidation and endothelial dysfunction. The atherogenic effects of Hcy are further compounded by its interaction with oxidative stress and lipid metabolism, particularly through the promotion of LDL oxidation. The oxidation of LDL to form ox-LDL represents a critical step in atherogenesis. Serum ox-LDL concentration reflects *in vivo* LDL oxidative stress and actively participates in atherosclerosis development (20). ox-LDL is known to promote arterial wall inflammation and lipid deposition, processes that drive plaque formation through receptor-mediated uptake by macrophages leading to foam cell formation, and by triggering a pro-inflammatory cascade. It is also implicated in cardiovascular disease, diabetes, obesity, and metabolic syndrome (MetS) (21–24).

Our findings align with the concept that subclinical hypothyroidism creates a pro-oxidant and pro-inflammatory milieu. Thyroxine acts as an antioxidant for LDL. Consequently, thyroxine deficiency renders LDL more susceptible to oxidation. Elevated levels of both ox-LDL and LDL accompany hypothyroidism, thus increasing the risk of atherosclerosis (25, 26). Furthermore, hypothyroidism leads to elevated ox-LDL content, thereby increasing the extent of LDL oxidation (27). Aged lipoproteins are inherently vulnerable to oxidation, accumulating lipid peroxidation products that enhance oxidative damage (28, 29). Substantial evidence confirms that various oxidized LDL forms, particularly ox-LDL, contribute significantly to atherosclerosis through multiple mechanisms (30, 31). Hypercholesterolemia, which is frequently associated with hypothyroidism, increases the risk of atherosclerosis. Similarly, a study by Linton MF et al. found significantly elevated circulating ox-LDL levels in untreated hypothyroid patients, suggesting its contribution to the atherosclerotic process in this condition (32). Notably, Hcy, ox-LDL, and TSH levels demonstrate significant positive correlations in hypothyroidism patients. Furthermore, elevated Hcy may promote thrombosis by enhancing platelet aggregation and coagulation factor activity (33, 34). Thus, the interplay between HHcy and elevated ox-LDL is hypothesized to create a synergistic effect, potentially accelerating endothelial damage and atherosclerotic plaque progression in SCH.

The independent associations observed between TSH, ox-LDL, and Hcy can be contextualized within the multifaceted vascular actions of thyroid hormones. Thyroxine (T4) and triiodothyronine (T3) exert profound effects on the cardiovascular system through both genomic and non-genomic pathways. At the genomic level, T3 regulates the expression of key hepatic enzymes and receptors involved in lipid metabolism, including the low-density lipoprotein receptor (LDL-R) and cholesterol 7α-hydroxylase (CYP7A1). A subclinical deficiency can thus directly impair LDL-C clearance and bile acid synthesis, contributing to dyslipidemia. Perhaps more pertinent to the observed pro-oxidant state are the rapid, non-genomic actions. Thyroid hormones promote endothelial nitric oxide (NO) synthase activity via the PI3K/Akt pathway, which is critical for maintaining vasodilation, anti-inflammatory, and anti-apoptotic vascular homeostasis. Consequently, even mild thyroid hormone deficiency, as in SCH, can induce endothelial dysfunction (35). This compromised endothelial state increases vascular permeability and adhesion molecule expression, creating a fertile environment for the infiltration and oxidation of LDL particles. The elevated Hcy levels further exacerbate this by promoting oxidative stress and reducing NO bioavailability. Therefore, the biomarker profile identified in our study (elevated ox-LDL and Hcy, decreased HDL-C) likely represents the integrated consequence of thyroid hormone-mediated endothelial dysfunction and perturbed lipid metabolism.

Beyond intrinsic hormonal levels, the patient’s iodine status is a critical environmental modifier of SCH trajectory and associated risk. The relationship between iodine intake and thyroid dysfunction is U-shaped. In iodine-deficient regions, SCH may stem from compensatory glandular activity, and iodine supplementation can improve thyroid function and lipid profiles. In contrast, in iodine-replete or excessive settings, excess iodine (particularly from iodinated contrast media, amiodarone, or supplements) can trigger or exacerbate autoimmune thyroiditis, leading to persistent or progressive SCH. Iatrogenic iodine exposure, such as during coronary angiography, is a recognized precipitant of thyroid dysfunction, especially in antibody-positive individuals (36). Thus, in clinical practice, assessing dietary history, geographic iodine sufficiency, and recent exposure to iodinated agents provides essential context for the etiology and prognosis of SCH. For high-risk patients (e.g., TPOAb-positive), monitoring thyroid function after planned iodine exposure may be prudent.

The biomarkers elevated in our cohort, ox-LDL and Hcy, may serve as functional links between SCH and CMR comorbidities. Ox-LDL is a direct driver of atherosclerosis, while hyperhomocysteinemia is associated with insulin resistance and renal impairment. From a public health perspective, future research should leverage large-scale electronic health records (EHR) and biobanks to systemically track the longitudinal co-occurrence and sequence of SCH, atherogenic dyslipidemia, elevated Hcy, and incident CMR diseases. Employing data mining techniques on such platforms could help identify high-risk phenotypic clusters and critical windows for preemptive intervention.

**Figure 4:**
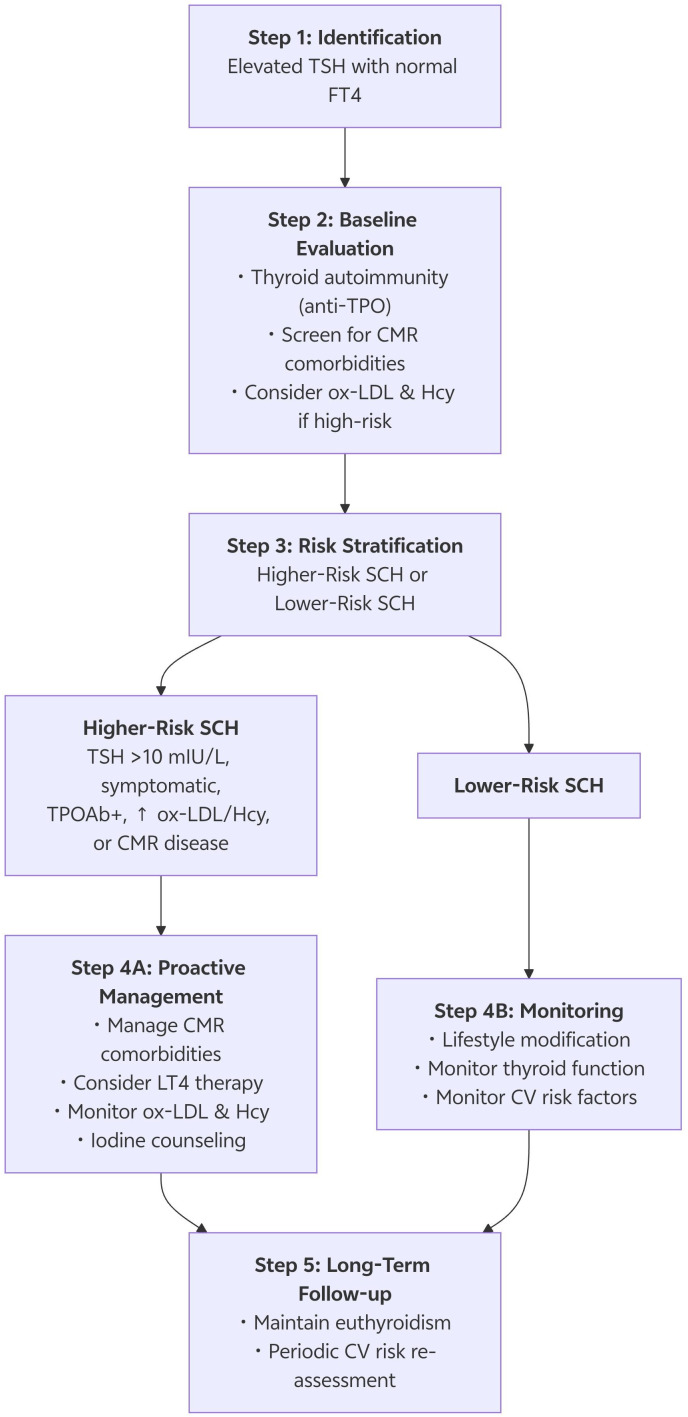
Proposed integrated management algorithm for adults with subclinical hypothyroidism. FT4, free thyroxine; CMR, cardio-metabolic-renal; TPOAb, anti-thyroid peroxidase antibody; LT4, levothyroxine; CV, cardiovascular.

The cardiovascular risk associated with SCH is not uniform. Evidence suggests a gradient of risk, with the following features marking higher-risk subgroups: (1) Higher TSH levels (e.g., >10 mIU/L); (2) Evidence of autoimmune thyroiditis (positivity for TPOAb or TgAb); (3) Co-existing CMR conditions (e.g., diabetes, hypertension); (4) Specific demographic factors, such as younger to middle age (<65 years, due to longer lifetime risk exposure), female sex, and individuals from regions with unstable iodine nutrition (37). These subgroups should be prioritized for more vigilant cardiovascular risk assessment and management. Synthesizing our findings with current evidence, we propose a practical, stepwise algorithm for the assessment and management of adults with subclinical hypothyroidism (SCH), integrating emerging biomarkers for refined risk stratification (see **Figure 4**). First, upon identification of SCH (elevated TSH with normal FT4), a comprehensive baseline evaluation is recommended, extending beyond thyroid function to include: (i) thyroid autoimmunity status (anti-TPO antibodies); (ii) screening for CMR comorbidities (blood pressure, fasting glucose, lipid profile, renal function); and (iii) consideration of ‘risk-enhancing’ biomarkers—measurement of oxidized low-density lipoprotein (ox-LDL) and homocysteine (Hcy) is particularly warranted in patients with TSH levels between 4–10 mIU/L who harbor one or more traditional risk factors. Subsequently, patients can be stratified into ‘Higher-Risk SCH’ (defined by the presence of any of the following: TSH >10 mIU/L, being symptomatic, TPOAb-positive, having elevated ox-LDL/Hcy, or established CMR disease) or ‘Lower-Risk SCH’. Based on this stratification, management is personalized: For Lower-Risk SCH, the emphasis should be on lifestyle modification and monitoring of thyroid function and conventional risk factors every 6–12 months. Conversely, for Higher-Risk SCH, a more proactive approach is advised, which includes: (a) initiating or intensifying management of CMR comorbidities (e.g., statin therapy); (b) engaging in shared decision-making regarding levothyroxine (LT4) therapy, with a lower treatment threshold for younger patients, those with TSH>10, or significant symptoms; (c) utilizing ox-LDL and Hcy as dynamic monitors of response to lifestyle or pharmacological interventions (e.g., LT4, statins); and (d) providing pre-emptive counseling regarding the risk of iodine-induced thyroid dysfunction prior to procedures using iodinated contrast media. Finally, long-term follow-up should focus on maintaining euthyroidism (TSH within an individualized target range) and on the periodic re-assessment of the global cardiovascular risk profile.

Our findings demonstrate that TSH levels are independently associated with ox-LDL and Hcy in both euthyroid subjects and those with SCH. Within the observed association between TSH and cardiovascular disease risk markers, ox-LDL and Hcy may represent important intermediary biomarkers. Elevated serum levels of TC, TG, and LDL-C are established indicators of increased cardiovascular risk. Thyroid hormones play a pivotal role in lipid metabolism regulation, and the elevation of TC, TG, and LDL-C is intrinsically linked to hypothyroidism. Furthermore, thyroid hormones are associated with endothelial dysfunction and other risk factors contributing to coronary artery disease (38, 39). The combined effect of hyperhomocysteinemia and hypercholesterolemia provides a mechanistic explanation for accelerated atherosclerosis in these patients. Notably, while other markers of oxidative stress (e.g., malondialdehyde, superoxide dismutase) have also been linked to thyroid dysfunction and CVD, ox-LDL represents a more specific and functionally central player in atherogenesis. Consequently, screening for ox-LDL and Hcy in hypothyroid patients warrants further investigation, as elevations in these markers are associated with an increased risk profile for atherosclerosis and cardiovascular disease. Furthermore, exploratory analyses indicated that the positive associations of TSH with ox-LDL and Hcy were consistent in direction for both men and women, with no statistically significant interaction effect by sex. This suggests that the pro-atherogenic biomarker profile linked to higher TSH levels may be pertinent across sexes, which is of clinical relevance given the higher prevalence of SCH in females. Our results are consistent with several studies in Western populations (9, 16) that reported similar associations, reinforcing the potential universality of these risk pathways, albeit within the context of our specific Chinese cohort.

This study has several potential limitations. First, the retrospective cross-sectional design precludes causal inference between TSH levels and cardiovascular risk factors; mechanistic studies are needed to establish causality. Second, although we adjusted for several clinical and metabolic confounders, we were unable to account for certain lifestyle and dietary variables—such as smoking, alcohol intake, physical activity, and micronutrient status (e.g., vitamins B6, B12, folate)—which are known to influence oxidative stress, homocysteine metabolism, and lipid profiles. The absence of these data may affect the observed associations and should be considered when interpreting our results. Third, the sample size of the SCH group (n=78) was smaller than that of the euthyroid subgroups. While this reflects the real-world prevalence of SCH, it may limit the statistical power for within-group comparisons and increase the risk of type II errors. Nevertheless, our primary analyses (e.g., correlation and multivariate regression) utilized the full cohort and were less susceptible to this imbalance. Fourth, our study involved multiple comparisons across several biomarkers, which increases the risk of Type I error. We applied the Benjamini-Hochberg FDR correction to address this concern. The core findings regarding ox-LDL, Hcy, and HDL-C remained statistically robust after correction. However, the associations for LDL-C and ApoB, while nominally significant, did not survive strict FDR correction, indicating the need for cautious interpretation and replication in larger cohorts. Finally, and importantly, our study lacked data on anti-thyroid antibodies (anti-TPO and anti-TG). A substantial proportion of SCH, particularly in iodine-sufficient regions, is attributable to autoimmune thyroiditis (Hashimoto’s thyroiditis). The chronic low-grade inflammation associated with autoimmunity, as well as potential direct immune-mediated effects on the vasculature, could independently influence oxidative stress, homocysteine metabolism, and lipid profiles. Consequently, the observed associations between TSH and cardiovascular risk markers (ox-LDL, Hcy) in our SCH group may reflect a confluence of effects from mild thyroid hormone deficiency and underlying autoimmune inflammation. Our inability to stratify SCH patients by antibody status prevents us from disentangling these potentially distinct contributions. Future prospective studies with larger SCH cohorts are warranted to compare antibody-positive and antibody-negative patients, in order to clarify the specific pathogenic mechanisms involved and to confirm these findings.

Despite the associative nature of our findings, they hold potential clinical relevance. The robust independent associations between TSH, ox-LDL, and Hcy suggest that these biomarkers could help identify SCH individuals at heightened cardiovascular risk, potentially enabling earlier and more targeted interventions. While routine screening for ox-LDL and Hcy in all SCH patients is not yet standard practice and requires further cost-effectiveness evaluation, our study supports their investigation in selected high-risk cases. Future research should focus on whether ameliorating these biochemical risk factors through thyroid hormone normalization or other therapies translates into improved cardiovascular outcomes.

## Conclusion

In conclusion, this cross-sectional study demonstrates associations among elevated TSH, increased levels of ox-LDL and Hcy, and dyslipidemia in adults with subclinical hypothyroidism and high-normal TSH. These findings suggest a pro-atherogenic state that may contribute to accelerated cardiovascular risk in this population. The independent associations of TSH with ox-LDL and Hcy highlight their potential role as mediating factors. Future prospective studies are needed to establish causality and determine the clinical utility of these biomarkers in risk stratification and management.

## Data Availability

The raw data supporting the conclusions of this article will be made available by the authors, without undue reservation.
