# Supplementary material for: The study on serum oxidized low-density lipoprotein and homocysteine as cardiovascular risk markers in subclinical hypothyroidism patients
Source: Front Endocrinol (Lausanne). 2026 Jan 23;17:1750486. doi: 10.3389/fendo.2026.1750486 (PMC12875961; doi:10.3389/fendo.2026.1750486)
Supplement: Supplementary file 1 [file Table1.docx]

Supplementary Table 1 Sex-Stratified Comparison of Cardiovascular Risk Factors Across TSH Subgroups

| Variable | Male (n=422) |  |  |  |  | Female (n=254) |  |  |  |  |
| --- | --- | --- | --- | --- | --- | --- | --- | --- | --- | --- |
|  | T1 (n=132) | T2 (n=121) | T3 (n=126) | SCH (n=43) | p-value | T1 (n=68) | T2 (n=79) | T3 (n=72) | SCH (n=35) | p-value |
| TC (mmol/L) | 4.65 ± 0.85 | 4.92 ± 0.98 | 4.88 ± 0.88 | 5.02 ± 1.05 | 0.08 | 4.92 ± 0.98 | 5.15 ± 1.12 | 5.03 ± 0.91 | 5.25 ± 1.18 | 0.15 |
| TG (mmol/L) | 1.65 (1.08-2.18) | 1.68 (1.10-2.10) | 1.55 (1.08-2.20) | 1.61 (1.05-2.22) | 0.35 | 1.48 (0.92-1.95) | 1.55 (0.95-1.85) | 1.42 (0.98-1.96) | 1.44 (0.88-2.01) | 0.41 |
| HDL-C (mmol/L) | 1.35 ± 0.32 | 1.34 ± 0.29 | 1.30 ± 0.40 | 1.22 ± 0.31 | <0.01 | 1.55 ± 0.40 | 1.52 ± 0.31 | 1.48 ± 0.44 | 1.38 ± 0.35 | <0.01 |
| LDL-C (mmol/L) | 2.58 ± 0.82 | 2.80 ± 0.83 | 2.89 ± 0.92 | 2.96 ± 0.79 | 0.02 | 2.53 ± 1.02 | 2.75 ± 0.96 | 2.86 ± 1.07 | 2.98 ± 0.94 | 0.04 |
| ApoA1 (g/L) | 1.22 ± 0.13 | 1.26 ± 0.15 | 1.30 ± 0.18 | 1.29 ± 0.14 | 0.38 | 1.27 ± 0.15 | 1.31 ± 0.17 | 1.35 ± 0.20 | 1.34 ± 0.16 | 0.51 |
| ApoB (g/L) | 0.91 ± 0.12 | 0.95 ± 0.16 | 0.97 ± 0.10 | 1.06 ± 0.14 | <0.01 | 0.93 ± 0.16 | 0.97 ± 0.20 | 1.00 ± 0.12 | 1.13 ± 0.18 | <0.001 |
| Lp(a) (mg/dL) | 12.8 (5.5-23.1) | 13.5 (6.5-20.8) | 14.5 (7.5-22.5) | 12.5 (7.8-25.0) | 0.81 | 12.1 (5.8-22.4) | 12.8 (7.2-19.5) | 13.8 (8.2-21.6) | 11.9 (8.1-23.9) | 0.69 |
| ox-LDL (ng/mL) | 1.10 ± 0.65 | 1.28 ± 0.55 | 1.49 ± 0.36 | 1.81 ± 0.48 | <0.001 | 0.98 ± 0.72 | 1.17 ± 0.57 | 1.38 ± 0.40 | 1.74 ± 0.50 | <0.001 |
| Hcy (μmol/L) | 9.45 (8.30-10.60) | 9.40 (8.45-11.05) | 9.65 (8.35-11.45) | 9.90 (8.40-11.30) | 0.03 | 8.85 (7.80-9.70) | 9.12 (8.15-10.75) | 9.28 (8.05-10.95) | 10.05 (8.55-12.15) | <0.01 |

**Abbreviations:** SCH, subclinical hypothyroidism; TC, total cholesterol; TG, triglycerides; HDL-C, high-density lipoprotein cholesterol; LDL-C, low-density lipoprotein cholesterol; ApoA1, apolipoprotein A1; ApoB, apolipoprotein B; Lp(a), lipoprotein(a); ox-LDL, oxidized low-density lipoprotein; Hcy, homocysteine.

**Notes**: Data are presented as mean ± standard deviation or median (interquartile range). p-values were derived from one-way ANOVA (for normally distributed data) or Kruskal-Wallis test (for non-normally distributed data) across the four TSH subgroups within each sex. The sample size for each sex-specific subgroup is indicated in the column headers.
